# Regulation of Wheat Seed Dormancy by After-Ripening Is Mediated by Specific Transcriptional Switches That Induce Changes in Seed Hormone Metabolism and Signaling

**DOI:** 10.1371/journal.pone.0056570

**Published:** 2013-02-20

**Authors:** Aihua Liu, Feng Gao, Yuri Kanno, Mark C. Jordan, Yuji Kamiya, Mitsunori Seo, Belay T. Ayele

**Affiliations:** 1 Department of Plant Science, University of Manitoba, Winnipeg, Manitoba, Canada; 2 Cereal Research Centre, Agriculture and Agri-Food Canada, Winnipeg, Manitoba, Canada; 3 RIKEN Plant Science Center, Tsurumi, Yokohama, Japan; Centro de Investigación y de Estudios Avanzados del IPN, Mexico

## Abstract

Treatments that promote dormancy release are often correlated with changes in seed hormone content and/or sensitivity. To understand the molecular mechanisms underlying the role of after-ripening (seed dry storage) in triggering hormone related changes and dormancy decay in wheat (*Triticum aestivum*), temporal expression patterns of genes related to abscisic acid (ABA), gibberellin (GA), jasmonate and indole acetic acid (IAA) metabolism and signaling, and levels of the respective hormones were examined in dormant and after-ripened seeds in both dry and imbibed states. After-ripening mediated developmental switch from dormancy to germination appears to be associated with declines in seed sensitivity to ABA and IAA, which are mediated by transcriptional repressions of *PROTEIN PHOSPHATASE 2C*, *SNF1-RELATED PROTEIN KINASE2*, *ABA INSENSITIVE5* and *LIPID PHOSPHATE PHOSPHTASE2*, and *AUXIN RESPONSE FACTOR* and *RELATED TO UBIQUITIN1* genes. Transcriptomic analysis of wheat seed responsiveness to ABA suggests that ABA inhibits the germination of wheat seeds partly by repressing the transcription of genes related to chromatin assembly and cell wall modification, and activating that of GA catabolic genes. After-ripening induced seed dormancy decay in wheat is also associated with the modulation of seed IAA and jasmonate contents. Transcriptional control of members of the *ALLENE OXIDE SYNTHASE*, *3-KETOACYL COENZYME A THIOLASE*, *LIPOXYGENASE* and *12-OXOPHYTODIENOATE REDUCTASE* gene families appears to regulate seed jasmonate levels. Changes in the expression of GA biosynthesis genes, *GA 20-OXIDASE* and *GA 3-OXIDASE*, in response to after-ripening implicate this hormone in enhancing dormancy release and germination. These findings have important implications in the dissection of molecular mechanisms underlying regulation of seed dormancy in cereals.

## Introduction

Seeds dormancy is an adaptive trait that blocks the germination of intact viable seeds under favourable conditions [1]. However, cereal crops such as wheat (*Triticum aestivum*) are selected for reduced seed dormancy to achieve rapid and uniform germination [2], resulting in increased susceptibility to preharvest sprouting (PHS) under wet field conditions prior to harvest. Since PHS causes substantial loss in seed yield and quality [3], there is a need to develop cultivars with an intermediate level of dormancy. This requires understanding of mechanisms underlying the maintenance and release of seed dormancy in cereal crops. Previous studies have demonstrated that seed dormancy and germination are regulated by interaction of plant hormones in both synergistic and competing manner, and treatments that promote dormancy decay such as after-ripening, which in wheat occurs after a period of dry storage conditions, are often correlated with changes in seed hormone content and/or sensitivity [4].

ABA regulates seed dormancy and germination [5]. Genetic mutations that cause ABA-deficiency promote seed germination, whereas those causing ABA accumulation enhance dormancy [6]. The seed ABA content is modulated by a balance between its biosynthesis and catabolism, which is regulated mainly by two ABA biosynthetic enzymes, zeaxanthin epoxidase (ZEP) and 9-cis-epoxycaretonoid dioxygenase (NCED), and an enzyme that catalyzes the predominant ABA catabolic pathway, ABA 8′-hydroxylase (ABA 8′OH) [6]. Genes encoding NCED play important roles in regulating seed ABA level and dormancy in developing seeds [7]–[9]. Loss of Arabidopsis (*Arabidopsis thaliana*) and barley (*Hordeum vulgare*) seed dormancy by after-ripening is associated with decreases in seed ABA content, which occurs mainly through transcriptional activation of specific members of *CYP707A* that encodes ABA 8′OH, and seed sensitivity to ABA [10]–[11]. However, wheat seed dormancy is related only to seed ABA sensitivity but not to ABA content [12]–[13]. Recent studies in Arabidopsis have shown that protein phosphatase 2C (PP2C) and SNF1-related protein kinase 2 (SnRK2) act as negative and positive regulators of ABA signaling, respectively [14]–[15]. Indeed, loss of function mutation in *PP2C* results in seed hypersensitivity to ABA whereas mutations in SnRK2s lead to strong ABA insensitivity [16]. The action of PP2C is controlled by ABA receptors, pyrabactin resistance (PYR)/PYR-like (PYL)/regulatory components of ABA receptors (RCAR), which upon binding to ABA form a complex with PP2C and inactivate it. Inactivation of PP2Cs leads to derepression of SnRK2, which activates the downstream transcription factors including ABA responsive element (ABRE) binding factor (ABF), ABA insensitive5 (ABI5), ABI3 and ABI4, and thereby mediate seed responsiveness to ABA [6]. Consistently, the triple (*pyr1pyl1pyl4*) and quadruple (*pyr1pyl1pyl2pyl4*) loss of function mutants of Arabidopsis exhibit strong insensitivity to ABA [17].

GA also regulates seed dormancy decay and germination [4]. For example, GA deficient seeds of Arabidopsis and tomato (*Lycopersicon esculentum*) are dormant and require GA to complete their germination [18]–[19]. After-ripening induced seed dormancy decay in dicot species is often correlated with increases in seed GA level and sensitivity [20]. In contrast, GA appears not to be involved in after-ripening mediated dormancy release in cereals [11], [21]. Although GA is not required for completion of cereal seed germination [22], it enhances the germination process through activation of hydrolytic enzymes such as α-amylase, and thereby storage reserve mobilization [23]. Components of GA signaling also play roles in regulating seed germination [24]. A mutation in the GA receptor, *GA INSENSITIVE DWARF1* (*GID1*) of rice (*Oryza sativa*) led to repression of the synthesis of α-amylase, although did not cause complete inhibition of germination [22]. DELLA proteins negatively regulate GA signaling and inhibit various GA responses including seed germination. Consistently, mutation in the barley DELLA gene, *SLENDER1* (*SLN1*), activates GA responsiveness in barley aleurone [25]. Activation of GA responses is mediated by degradation of DELLA, which requires SLEEPY1 (SLY1) [26]–[27]. The SPINDLY (SPY) protein on the other hand represses GA signaling [28]. Mutations in *SPY* and *SLY* have been shown to affect seed germination in Arabidopsis [29]–[30].

Other plant hormones such as jasmonate and auxin are also implicated in regulating seed dormancy and germination [20]–[31]. Jasmonate stimulates the germination of dormant seeds in *Acer tataricum* [32] and apple (*Malus domestica*) [33]. Furthermore, the expression of jasmonate biosynthetic genes including *LIPOXYGENASE6* (*LOX6*), *12-OXOPHYTODIENOATE REDUCTASE3* (*OPR3*) and *ALLENE OXIDE SYNTHASE* (*AOS*), and the seed jasmonic acid (JA) and JA-isoleucine (JA-Ile) content have been shown to be associated with maintenance or loss of seed dormancy [11], [34]. The action of jasmonate takes place via *CORONTAINE INSENSITIVE1* (*COI1*), whose expression is induced by after-ripening in barley [11]. Conversely, mutations in *COI1*, and *JASMONATE INSENSITIVE4* (*JIN4*), another jasmonate signaling factor, lead to seed hypersensitivity to ABA [35]–[36]. These results indicate the antagonistic effect of jasmonate to ABA-mediated inhibition of germination. However, the mechanism linking jasmonate to seed dormancy release and germination is still unclear. Recent studies have shown that seed indole acetic acid (IAA) level increases with imbibition of non-dormant seeds [34], [37], suggesting its importance in regulating seed germination, and dormant seeds contain over twofold less IAA than non-dormant seeds [34]. However, exogenous auxin increases seed ABA sensitivity and thereby enhances inhibition of germination [31], [38]. Consistently, derepression of *AUXIN RESPONSE FACTOR* (*ARF*), a mediator of plant response to auxin, increased seed ABA sensitivity and inhibition of germination [31], and auxin-resistant mutants exhibit reduced seed dormancy [39]. However, it is unclear if auxin mediates seed dormancy release during after-ripening.

Although previous studies, mainly with dicot species, have provided insights into the involvement of several plant hormones in regulating seed dormancy and germination through a variety of synergistic and antagonistic interactions [40]. This phenomenon is poorly understood in wheat. To gain insights into the role of plant hormones in regulating after-ripening mediated seed dormancy decay and its subsequent germination in wheat and identify related marker genes, we performed comprehensive analysis of the temporal expression patterns of metabolic and signalling genes of ABA, GA, jasmonate and auxin, and the respective hormone levels in dormant and after-ripened seeds in both dry and imbibed states. Furthermore, using large scale gene expression analysis, we assessed the physiological and metabolic states of wheat seed responsiveness to ABA during imbibition.

## Materials and Methods

### Plant Materials and Growth Conditions

Seeds of *Triticum aestivum* (L.) cv. AC Domain were used for this study. AC Domain is hard red spring wheat that exhibited a high level of PHS tolerance and is adapted to the Canadian prairies [41]. Plant growth conditions, seed harvesting and generation of after-ripened seeds are described previously [42].

### Germination Assay and ABA Treatment

For germination, transcriptome and hormone level analysis, the dormant and after-ripened seeds of cv. AC Domain were surface sterilized and imbibed in water for 12 and 24 h as described before [42]. To investigate the effect of ABA on germination and gene expression during imbibition, the same AR seed sample used in the previous study was imbibed with 50 µM ABA for 24 h. Imbibed seeds were harvested in liquid N_2_ and stored at -80°C until further use. Unimbibed dormant and after-ripened seeds were used for gene expression and hormone level analysis in dry seeds. Further comparison of germination and seminal root growth was performed with dormant and after-ripened seeds (with or without ABA) imbibed for 36 and 48 h.

### RNA Isolation and Microarray Analysis

RNA isolation and microarray analysis were performed as described before [42]. Briefly, mRNA was isolated from three independent biological replicates of dry dormant and after-ripened seeds, dormant and after-ripened seeds imbibed in water for 12 and 24 h, and after-ripened seeds imbibed in ABA for 24 h. The mRNA samples were labeled and hybridized to the Affymetrix GeneChip Wheat Genome Array (Affymetrix). After washing and scanning of the hybridized microarrays, the data from the 11 probe pairs were converted into a single hybridization intensity level per probeset using the Affymetrix GeneChip Operating Software and then represented in CEL file format. The number of probesets with a “present” detection call in each sample was determined by the Affymetrix Microarray Suite (MAS5) statistical algorithm. Verification of the reproducibility of the data derived from the three independent biological replicates was performed by scatter plot expression analysis. Robust Multi-array Average (RMA) methodology was used to normalize the raw intensity data, which was then logarithmically (base 2, log_2_) transformed. The microarray dataset discussed in this paper has been deposited in NCBI Gene Expression Omnibus database (GSE32409). Validation of the microarray data with qPCR is as described before [42].

### Identification of Wheat Probesets Related to Hormone Metabolism and Signaling

Genes involved in ABA, GA, jasmonate and IAA metabolism and signalling were identified from Arabidopsis, rice and other monocot species using publicly available gene index databases. The target sequences identified from the dicot plant Arabidopsis were first subjected to sequence similarity searches against the Rice Annotation Project database (http://rapdb.dna.affrc.go.jp/) [43] using a criterion of E-value of <10^−20^. To identify related genes in wheat, similarity searches were performed with the respective sequences from rice and other monocot species against the NCBI wheat unigene dataset containing 56,954 unigenes (http://www.ncbi.nlm.nih.gov/UniGene/UGOrg.cgi?TAXID=4565) [44] using the criteria of ≥200 bp coverage length and E-value of ≤10^−50^. The resulting wheat EST or cDNA sequences were blasted against the wheat 61 k microarray platform using the Plant Expression Database (PLEXdb) Blast (http://www.plexdb.org/modules/PD_general/tools.php) [45] to identify the corresponding probesets on wheat GenChip with E-value of ≤10^−50^. Annotation of the candidate probesets shown in Table S1 was performed using HarvEST Wheat-Chip (http://harvest.ucr.edu) [46].

### Expression Analysis of Hormone Metabolism and Signaling Related Wheat Probesets

Log_2_ transformed signal intensities of probesets corresponding to ABA, GA, jasmonate and IAA metabolism and signalling genes were extracted from the microarray dataset described above (Table S1) and presented in log_2_ scaled fold change between imbibition time points (0 h, 12 h and 24 h) within each seed sample (dormant and after-ripened), and between the two seed samples at each imbibition time point, along with the associated *P* values (Table S2). Expression values are also given in linear scaled fold changes (Table S2). Negative and positive fold changes indicate downregulation and upregulation of expression in each comparison, respectively. Analysis of the data was performed by FlexArray software (http://genomequebec.mcgill.ca/Flex-Array) [47] using analysis of variance (ANOVA) as described before [42]. To reduce any variation due to technical factors, only hormone metabolism and signaling probesets with ≥2-fold change in expression and a probability value of *P*≤0.05 were considered to exhibit statistically significant differential expression. Heat maps of the identified probesets were generated from expression values in log_2_ fold change by MultiExperiment Viewer (MeV version 4.6) [48]. Comparative analysis of after-ripening and GA regulated genes was performed by translating wheat probesets into their Arabidopsis and barley equivalents using the microarray platform translator (http://www.plexdb.org/modules/PD_general/tools.php) [45]. AgriGO analysis toolkit (http://bioinfo.cau.edu.cn/agriGO/analysis.php) [49] and HarvEST WheatChip (http://harvest.ucr.edu/) [46] were used to predict GO for each probeset and annotate the candidate genes, respectively.

### Expression Analysis of *TaGA3ox2* Gene

Primers for *TaGA3ox2* (5′-GACTCGGGCTTCTTCACCTT-3′ and 5′-TGGTGAGGATCTGGAAGAGG-3′) were designed from the conserved regions of cDNA sequences derived from the three genomes of hexaploid wheat (GenBank IDs: DQ118250, DQ118251, DQ118252) [50], whereas those for actin (5′- GCTGGAAGGTGCTGAGGGA-3′ and 5′-GCATCGCCGACAGGATGAG-3′) were designed based on the reported sequence of Taβactin (GenBank ID: CD899716; unpublished). Primer specificity was determined by blasting primer and amplicon sequences against GenBank database and RT-PCR. Real time qPCR analysis was performed with cDNAs prepared from the same mRNA samples used for microarray analysis and an EvaGreen two-step qPCR Supermix on CFX96 real-time PCR system (Bio-Rad). The qPCR reaction and thermal cycling conditions are described previously [51]. Transcript levels of *TaGA3ox2* were expressed after normalization with actin as previously described [52].

### Hormone Quantification

Seed hormone levels were quantified from three independent biological replicates (400–800 mg dry weight per replication) of lyophilized air-dry (0 h after imbibition [HAI]) and water imbibed samples (12 and 24 HAI) of the same dormant and after-ripened seeds used for microarray and germination analysis. Quantification of seed hormone content was repeated for both dormant and after-ripened samples using another independently grown seed batch (2–3 biological replicates per sample) except for the 12 HAI samples. Extraction and purification of ABA, JA, JA-Ile and IAA were performed as described previously [53], except that the extraction was performed with 10 ml of 80% (v/v) acetonitrile containing 1% (v/v) acetic acid. Quantification of the seed hormone levels was carried out with LC-ESI-MS/MS system as described before [54].

Significant differences between samples for all non-microarray data noted in the text were also tested by ANOVA using a probability of *P*≤0.05.

## Results and Discussion

### Germination Performance of Dormant and After-ripened Seeds

**Figure 1:**
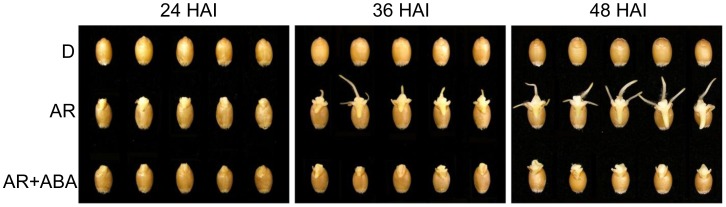
Imbibed dormant (D) and after-ripened (AR) seeds of wheat cv. AC Domain. Seeds imbibed in water (D and AR) and 50 µM ABA (AR) at 24, 36 and 48 h after imbibition (HAI).

The germination performance of the dormant and after-ripened seeds has been reported previously [42]. Water imbibed after-ripened seeds germinated (coleorhiza emergence through the seed coat) after 24 h whereas imbibing the after-ripened seeds with ABA (50 µΜ) delayed their germination (coleorhiza emergence through the seed coat) until after 36 h (Table 1, Figure 1), when seminal roots were observed in water imbibed after-ripened seeds. No seminal root was observed in ABA treated after-ripened seeds even after 48 h imbibition (Figure 1).

**Table 1:** Percentage germination of dormant and after-ripened seeds of wheat cv. AC Domain imbibed in water and ABA solution.

Sample	24 HAI	36 HAI	48 HAI
D	0	0	0
AR	99%±1.0^a^	100%±0.0	100%±0.0
AR+ABA^b^	4%±2.0	96%±2.4	100%±0.0

a Data are means ± SE, n = 3 (n refers to a batch of 25 seeds).

b Seeds were imbibed with 50 µM ABA solution.

### After-ripening Appears not to Alter ABA Metabolism in Wheat Seeds

**Figure 2:**
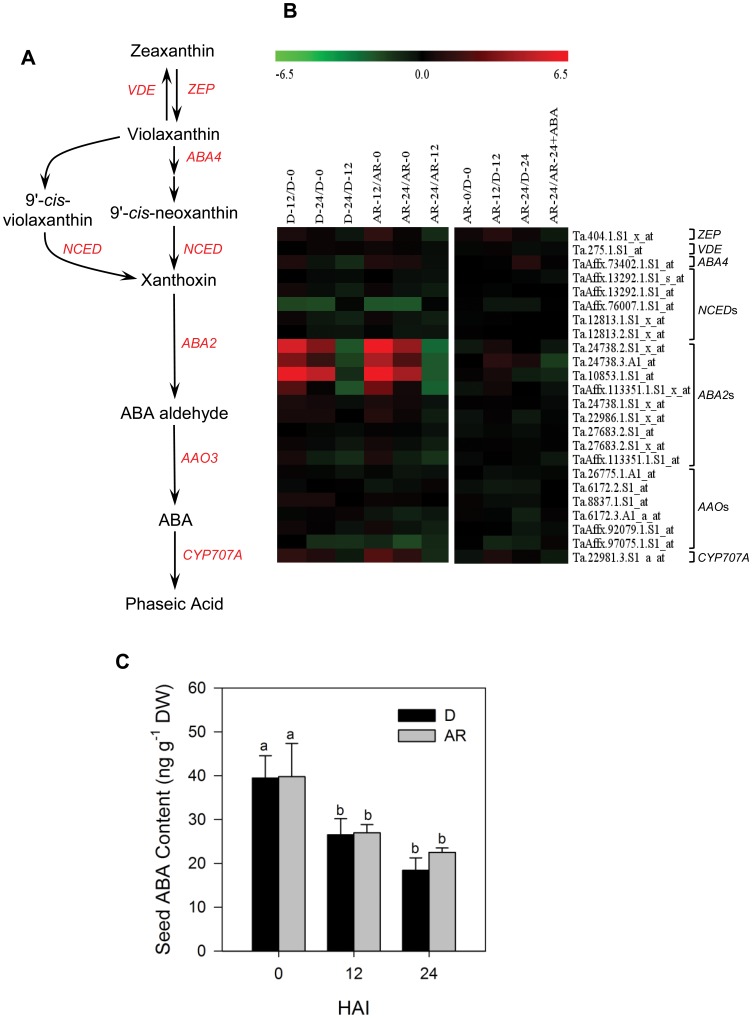
Comparison of the transcript abundance of abscisic acid (ABA) metabolic genes and seed ABA content. The ABA metabolism pathway in plants (A). Expression of probesets annotated as ABA metabolic genes in log_2_ fold change during imbibition of dormant (D-12/D-0, D-24/D-0 and D-24/D-0) and after-ripened (AR-12/AR-0, AR-24/AR-0 and AR-24/AR-0) seeds as shown in the first column of the heat map, between dormant and after-ripened seeds in both dry and imbibed states (AR-0/D-0, AR-12/D-12 and AR-24/D-24) and between water and ABA imbibed after-ripened seeds (AR-24/AR-24+ABA) as shown in the second column in each heat map (B). Log_2_ transformed signal intensities of the respective probesets were extracted from the microarray datasets (see Materials and Methods) and converted to expression values in log_2_ fold changes (the negative and positive numbers on the bar) shown by the color scale at the top of each heat map; higher and lower expression levels of the respective probesets are represented by red and green colors, respectively. Log_2_ and linear scaled fold changes in expression of the probesets and the respective *P* values can be found in Table S2. ABA content of D and AR seeds in dry (0 days after imbibition [HAI]) and imbibed (12 and 24 HAI) states (C). Data are means of ABA measurements from three independent biological replicates ± SE. Different letters between imbibition time points and between seed samples within each imbibition time indicate statistically significant difference in seed ABA level at *P*≤0.05. ZEP (ABA1), zeaxanthin epoxidase; VED, violaxanthin de-epoxidase; ABA4 (NSY), neoxanthin synthase; NCED, 9-cis-epoxycarotenoid dioxygenase; ABA2, ABA deficient 2 (alcohol dehydrogenase); AAO, abscisic aldehyde oxidase; CYP707A; ABA 8′-hydroxylase.

Seed ABA content is regulated by the balance between its biosynthesis and catabolism [6]. Genes encoding ABA metabolic enzymes, ZEP (ABA1), violaxanthin de-epoxidase (VDE), neoxanthin synthase (ABA4/NSY), NCED, ABA deficient2 (ABA2), abscisic aldehyde oxidase (AAO) and CYP707A (Figure 2A), have been identified from several plant species. Analysis of our dataset showed the presence of one probeset representing each of *ZEP*, *VDE* and *ABA4* on wheat GeneChip exhibiting no differential expression between dormant and after-ripened samples, except that the probeset annotated as *ZEP* was upregulated (2-fold, *P*≤0.05) in 12 h imbibed after-ripened relative to the corresponding dormant seeds (Figure 2B, Table S2). Specific members of the *NCED* family such as *NCED6* and *9* of Arabidopsis and *NCED2* of barley control ABA level and dormancy in developing seeds [7]–[8], and the expression of *NCED1* is closely associated with ABA level in imbibing dormant seeds of Brachypodium (*Brachypodium distachyon*) [55]. One of the five wheat probesets annotated as *NCED* exhibited over 3-fold downregulation (*P*≤0.05) upon imbibition in both dormant and after-ripened seeds, while the other four maintained constant expression (Figure 2B, Table S2) [42]. Similarly, one of the six probesets annotated as *AAO* exhibited over 2-fold downregulation (*P*≤0.05) in both seed samples following 24 h imbibition (Figure 2B, Table S2). In contrast, four out of the nine probesets representing *ABA2* exhibited upregulation (4.7- to 86.6-fold, *P*≤0.05) in imbibing seeds of both dormant and after-ripened samples (Figure 2B, Table S2). The ABA catabolic *CYP707A* genes have also been shown to regulate seed dormancy through modulating ABA content. For example, in barley and Brachypodium, *CYP707A1* expression is upregulated during imbibition of after-ripened seeds [55]–[56]. However, the expression of the only probeset representing *CYP707A* was found to be similar between dormant and after-ripened seeds [42], although its expression was induced (2.4-fold in dormant and 4.7-fold in after-ripened, *P*≤0.05) during the first 12 h of imbibition in both seed samples (Figure 2B, Table S2). These results indicate that specific members of the *NCED*, *ABA2*, *AAO* and *CYP707A* family are regulated by imbibition but not by after-ripening.

To determine if the expression of ABA biosynthetic and catabolic genes is correlated with seed ABA and dormancy level, we measured the level of ABA in both dormant and after-ripened seeds before and after imbibition, and no significant difference in ABA content was evident between the two seed samples in both dry and hydrated states (Figure 2C). However, seed ABA content significantly declined (1.5-fold, *P*≤0.05) in both seed samples within the first 12 h of imbibition, exhibiting a close association with the expression of specific *NCED* and *CYP707A* genes. The upregulation of *ZEP* probeset upon imbibition of after-ripened seeds while ABA level exhibited a similar decline as observed in the corresponding dormant seeds might suggest that the expression of *ZEP* is post-transcriptionally controlled. In summary, our combined seed ABA content and gene expression data clearly indicate that after-ripening induces changes in wheat seed dormancy status without altering the dynamics of ABA metabolism.

### After-ripening Alters the Expression of Specific ABA Signaling Genes

**Figure 3:**
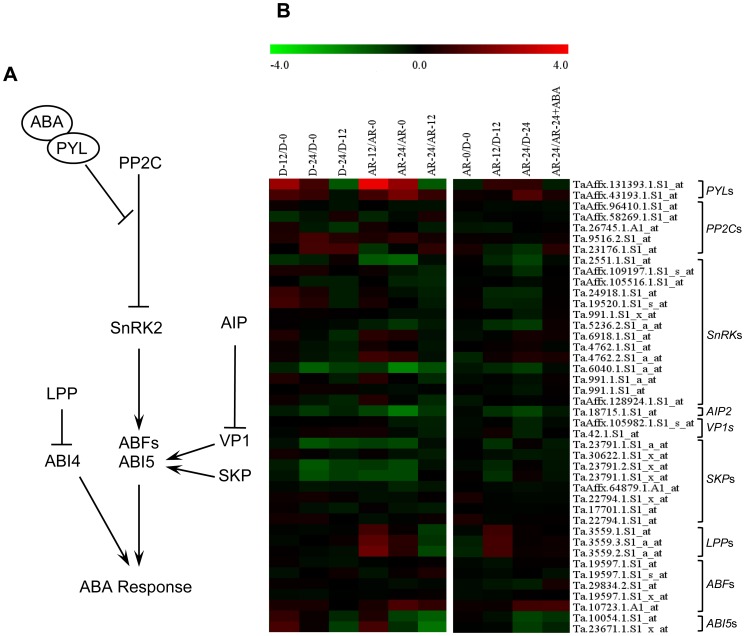
Comparison of the transcript abundance of abscisic acid (ABA) signaling genes. A model for ABA signaling pathway in plants (A). Expression of probesets annotated as ABA signaling genes in log_2_ fold change during imbibition of dormant (D-12/D-0, D-24/D-0 and D-24/D-0) and after-ripened (AR-12/AR-0, AR-24/AR-0 and AR-24/AR-0) seeds as shown in the first column of the heat map, between dormant and after-ripened seeds in both dry and imbibed states (AR-0/D-0, AR-12/D-12 and AR-24/D-24) and between water and ABA imbibed after-ripened seeds (AR-24/AR-24+ABA) as shown in the second column in each heat map (B). Determination of the fold changes in expression of each probeset is as described in Figure 2. Log_2_ and linear scaled fold changes in expression of the probesets and the respective *P* values can be found in Table S2. PYL, pyrabactin resistance like; PP2C, protein phosphatase 2C; SnRK, SNF1-related protein kinase2; AIP, ABI3-interacting protein 2; ABF, ABA responsive element binding factor; LPP; lipid phosphate phosphatase; SKP, S-phase kinase-associated protein; ABI3/4/5, ABA insensitive 3/4/5.

Previous studies have shown that seed dormancy in wheat is associated with seed ABA sensitivity [12]–[13]. To identify specific ABA signaling components that are involved in after-ripening mediated seed dormancy decay in wheat, we compared the expression of 63 probesets annotated as genes related to ABA signaling between after-ripened and dormant seeds in both dry and imbibed states. Members of the ABA receptor *PYR/PYL/RCAR* family form a complex with ABA to induce ABA-response [57]. Consistently, seeds from plants overexpressing *PYL8*/*RCAR3* exhibit increased ABA sensitivity and enhanced dormancy [58], while those derived from loss of function mutants exhibit strong ABA insensitivity [17]. One of the two probesets annotated as *PYL* genes exhibited upregulation (2.4-fold, *P*≤0.05) in after-ripened relative to dormant seeds following 24 h imbibition, while the other one showed a similar expression between the two seed samples (Figure 3A, Table S2). Since the ABA-responsive genes have been shown to be downregulated in imbibing after-ripened seeds [42], it is likely that these *PYL*s are either posttranscriptionally regulated or do not function as activators of ABA signaling in wheat seeds. The ABA-receptor complex binds to and represses PP2Cs [17], [59], several of which inhibit the SnRK2s [60] that positively regulate the downstream ABF and ABI5 bZIP transcription factors, and thereby induce seed ABA sensitivity and dormancy [61]. For example, the SnRK PKABA1 of wheat activates ABF, and thereby the transcription of ABA responsive genes [62]. While three of the five *PP2C* probesets displayed similar expression between dormant and after-ripened seeds, the remaining two probesets were upregulated (2.1-fold, *P*≤0.05) in imbibing dormant but not in after-ripened seeds (Figure 3A, Table S2). Similarly, all the *SnRK2* probsets were expressed equally in both dormant and after-ripened samples, except that one probeset showed downregulation (2-fold, *P*≤0.05) in 24 h imbibed after-ripened relative to the corresponding dormant seeds. These results might suggest that specific members of the *PP2C* and *SnRK2* genes of wheat are involved in the regulation of after-ripening induced loss of seed ABA sensitivity and dormancy.

Genetic studies in Arabidopsis have shown that ABI3, ABI4 and ABI5 are key mediators of seed response to ABA [63]. Two *ABI5* probesets were found on wheat GeneChip, and one of them showed downregulation (2.1-fold, *P*≤0.05) in 24 h imbibed after-ripened relative to dormant seeds (Figure 3B, Table S2). Although mutation in *ABI5* does not affect seed dormancy in Arabidopsis [64], the reduced dormancy and ABA sensitivity observed in the embryos of wheat mutant RSD32 is associated with repression of *TaABF*, the wheat homolog of *ABI5* [65]. Furthermore, higher expression of *ABI5* homolog and its corresponding protein has been reported in sorghum (*Sorghum bicolor*) cultivars with higher levels of dormancy [66]. Thus, the downregulation of one of the *ABI5* probesets in after-ripened wheat seeds might suggest its significance in regulating after-ripening mediated decline in seed ABA sensitivity and dormancy. Mutation in the *ABI3* orthologue of cereals, *VIVIPAROUS1* (*VP1*) [67], leads to the production of seeds susceptible to PHS [68]. Consistently, the expression of *VP1* is positively correlated with the level of seed dormancy in wild oats [69] and wheat [70]. However, the expression of *VP1* probesets remained unaffected by after-ripening (Figure 3B, Table S2). Since the VP1 protein is targeted for ubiquitination by a RING finger E3 ligase, ABI3-interacting protein2 (AIP2), during imbibition [71], the greater downregulation of *AIP2* probeset in imbibing after-ripened than dormant seeds (2.1-fold, *P*≤0.05) may suggest accumulation of VP1, and thereby activation of ABA responsive genes in after-ripened seeds. Contrary to this hypothesis, ABA responsive probesets are repressed in non-dormant embryos/after-ripened seeds of wheat [42], [72]. This could be due to the fact that the level of VP1 cannot be directly related to ABA sensitivity as wheat *VP1* is regulated by missplicing and forms little functional protein [73].

Phosphatidic acid, a lipid signaling molecule known to act upstream of ABI4, triggers ABA signal transduction related events during seed germination. The synthesis of phosphatidic acid is catalysed by lipid phosphate phosphatase2 (LPP2), and mutational analysis has shown that LPP2 represses seed sensitivity to ABA [74], and after-ripening activates the transcription of specific *LPP* genes in both Arabidopsis and barley [11], [75]. Similarly, all three probesets annotated as *LPP2* were upregulated (2-fold, *P*≤0.05) during the first 12 h imbibition in after-ripened relative to dormant seeds (Figure 3B, Table S2), suggesting that *LPP* represents one of the conserved mechanisms underlying after-ripening mediated loss in seed ABA sensitivity and dormancy. Although the S-phase kinase-associated protein1 (SKP1) positively regulates ABA signaling, and thereby induces seed hypersensitivity to ABA and dormancy [76], all the eight probesets annotated as *SKP1* showed no differential expression between dormant and after-ripened seeds in both dry and hydrated states.

### Transcriptomic Analysis of Wheat Seed Responsiveness to ABA

**Figure 4:**
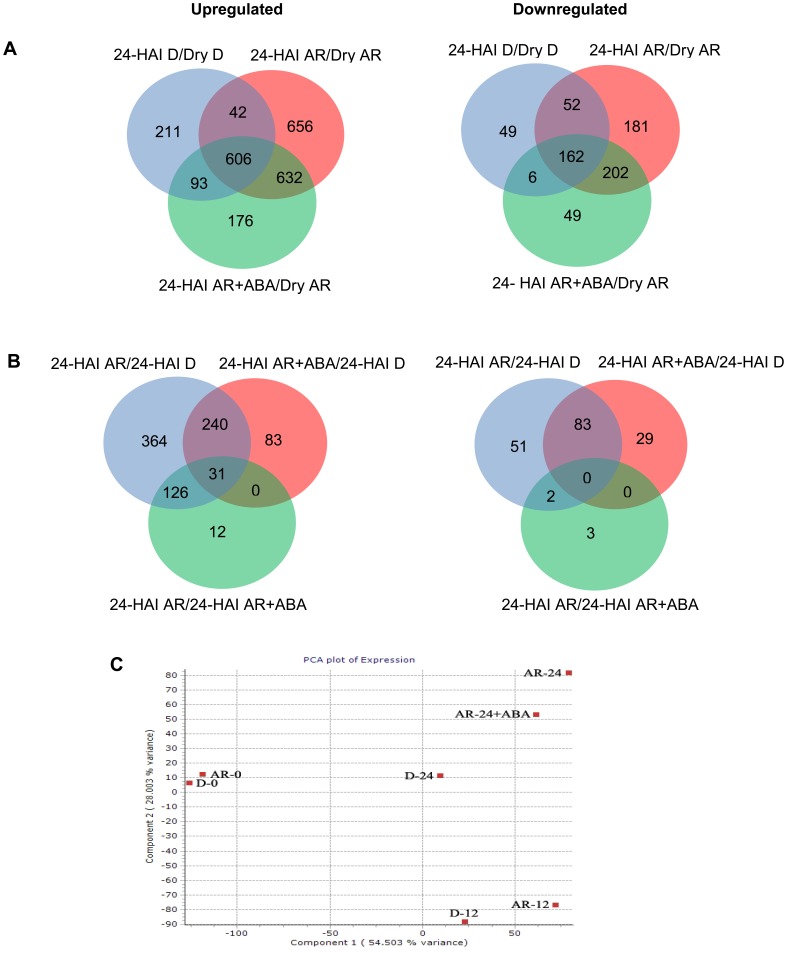
Probesets differentially expressed in dormant (D) and after-ripened (AR) seeds in dry and imbibed states. Comparisons of imbibed/dry (24 HAI D/Dry D, 24 HAI AR/Dry AR and 24 HAI AR+ABA/Dry AR) and imbibed/imbibed (24 HAI AR/24 HAI D, 24HAI AR+ABA/24 HAI D, 24 HAI AR/24 HAI AR+ABA) samples of D and AR seeds (A, B). The Venn diagrams show the number of significantly upregulated and downregulated probesets in each comparison (cutoff values fourfold change and *P*≤0.05). Probesets regulated in common are shown by the overlapping/intersecting region. Principal component analysis applied to the transcriptome dataset derived from seven samples (C); dry dormant seeds (D-0), dormant seeds imbibed in water for 12 (D-12) and 24 (D-24) h; dry after-ripened seeds (AR-0), after-ripened seeds imbibed in water for 12 (AR-12) and 24 (AR-24) h, and after ripened seeds imbibed for 24 h in 50 µM ABA (AR-24+ABA).

Comparison of changes in gene expression between water imbibed (24 h) after-ripened/dry after-ripened and water imbibed (24 h) dormant/dry dormant seeds revealed upregulation of 1288 (656+632) and downregulation of 383 (181+202) probesets in after-ripened seeds (at fourfold cutoff and *P*≤0.05; Figure 4A), suggesting the importance of genes represented by these probesets in regulating dormancy release by after-ripening in wheat. Nearly half of the probesets in each category (656 of the upregulated and 181 of the downregulated) are found specifically in water imbibed after-ripened seeds, indicating that the expression of genes represented by these probesets is controlled by ABA. Ontological analysis revealed that the 656 probesets whose expression is repressed by ABA are related to chromatin assembly (GO: 0031497, *P* = 3.4e−20), carbohydrate metabolic process (GO: 0005975, *P* = 2.2e−07) and cytoplasmic membrane-bound vesicle (GO: 0016023, *P* = 7.3e−07; Table S3); whereas the 181 probesets whose expression is induced by ABA are overrepresented in nutrient reservoir activity (GO: 0045735, *P* = 1.22e−06; Table S3). Twenty five of the ABA repressed probesets represent histone proteins, reflecting the role of ABA in regulating histone modification and chromatin assembly, processes implicated in seed germination [77]–[78]. These results imply that ABA’s role in delaying the germination of after-ripened seeds and inhibiting the growth of seminal root is associated with its repression or activation of the respective biological processes, which have been shown to be associated with after-ripening mediated seed dormancy decay and germination in wheat [42]. Consistently, the effect of ABA in inhibiting or delaying the germination of non-dormant seeds of Arabidopsis and barley, respectively, has been associated with ABA induced changes in gene transcription [11], [79].

Our analysis also showed up and downregulation of 632 and 202 probesets, respectively, that are common to both water and ABA imbibed after-ripened seeds (Figure 4A), suggesting that not all genes that control germination are regulated by ABA. The 632 upregulated probesets are related to cytoplasmic membrane-bound vesicle (GO: 0016023; *P* = 4.3e−16), DNA-dependent DNA replication (GO: 0006261; *P* = 7.1e−05), jasmonate metabolism (GO: 0009694; *P* = 9.6e−05) and α-amylase activity (GO: 0004556; *P* = 4.8e−06), while the 202 downregulated probesets are enriched in GO class of response to ABA stimulus (GO: 0009737; *P* = 1.5e−13; Table S4). Biological processes related to all these GO classes are implicated in seed germination [42], and could account for the inability of ABA treatment to completely restore dormancy phenotype in imbibing after-ripened seeds. Further analysis of seeds imbibed for 24 h revealed that over 2.6-fold more probesets (466/174) exhibit differential expression between ABA imbibed after-ripened and water imbibed dormant seeds than between ABA and water imbibed after-ripened seeds (at fourfold cutoff and *P*≤0.05; Figure 4B), suggesting that the physiological state of ABA imbibed after-ripened seeds mimics more of water imbibed after-ripened than dormant seeds. This is further confirmed by our Principal Component Analysis applied to the transcriptomic datasets derived from the seven samples (Figure 4C). Consistently, exogenous ABA was unable to mimic inherent dormancy in developing [40] and imbibing after-ripened seeds [75].

KaPPA-view based analysis of our datasets further revealed that imbibition of after-ripened seeds in ABA led to transcriptional repression (over 2-fold, *P*≤0.05) of probesets annotated as cell wall loosening (*xyloglucan endotransglycosylase7* and *basic chitinase*), starch and maltose degrading (*α-amylase* and *α-glucosidase*), and *glucosyl hydrolase* genes (Table S5), suggesting that ABA inhibits seed germination and seedling growth partly by suppressing embryo axis expansion and storage reserve mobilization. Consistently, these probesets exhibit upregulation in imbibed after-ripened relative to dormant seeds of wheat [42] and cell wall loosening genes are overrepresented in after-ripened than dormant seeds of Arabidopsis [80].

### After-ripening Activates the Transcription of GA Biosynthesis Genes during Imbibition

**Figure 5:**
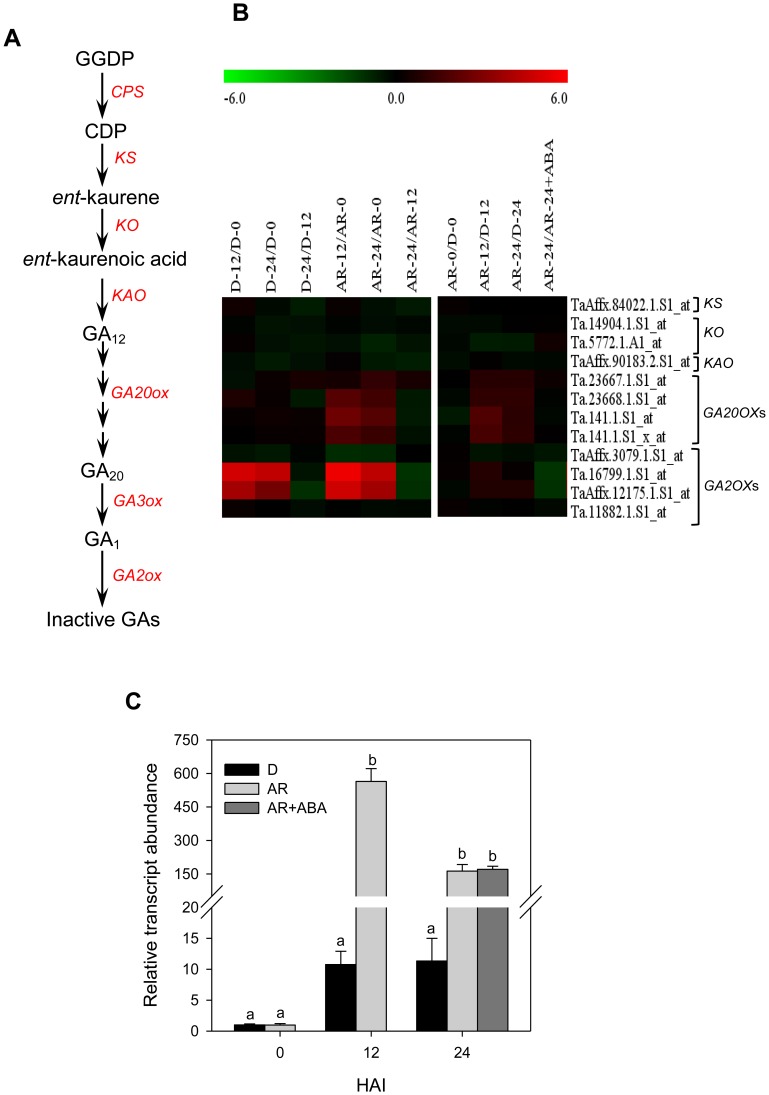
Comparison of the transcript abundance of gibberellin (GA) metabolic genes. The GA metabolism pathway in plants (A). Expression of probesets annotated GA metabolic genes in log_2_ fold change during imbibition of dormant (D-12/D-0, D-24/D-0 and D-24/D-0) and after-ripened (AR-12/AR-0, AR-24/AR-0 and AR-24/AR-0) seeds as shown in the first column of the heat map, between dormant and after-ripened seeds in both dry and imbibed states (AR-0/D-0, AR-12/D-12 and AR-24/D-24) and between water and ABA imbibed after-ripened seeds (AR-24/AR-24+ABA) as shown in the second column in each heat map (B). Determination of the fold changes in expression of each probeset is as described in Figure 2. Log_2_ and linear scaled fold changes in expression of the probesets and the respective *P* values can be found in Table S2. Relative transcript level of *TaGA3ox2* in D-0, D-12 and D-24, and AR-0, AR-12 AR-24 and AR-24+ABA wheat seeds (C). Transcript level was determined using Taβactin as the reference gene, and then expressed relative to that in D-0 seeds, which was arbitrarily set to a value of 1. Data are means of 2 to 3 independent biological replicates ± SE. Different letters between seed samples within each imbibition time indicate statistically significant difference in transcript abundance at *P*≤0.05. GGDP, geranyl geranyl diphosphate CDP, *ent*-copalyl diphosphate; CPS, ent-copalyl diphosphate synthase KS, ent-kaurene synthase KO, ent-kaurene oxidase KAO ent-kaurenoic acid oxidase GA20ox, gibberellin 20 oxidase GA3ox, gibberellin 3 oxidase; GA2ox, gibberellin 2 oxidase.

GA is associated with dormancy release and enhancement of seed germination [20]. Twelve probesets annotated as GA metabolism genes are found on wheat GeneChip, including *ENT-KAURENE SYNTHASE* (*KS*), *ENT-KAURENE OXIDASE* (*KO1*), *ENT*-*KAURENOIC ACID OXIDASE* (*KAO1*), *GA 20-OXIDASE* (*GA20ox1*) and *GA 2-OXIDASE* (*GA2ox1* and *8*; Figure 5A). Three of the four probesets representing *GA20ox1* exhibited upregulation (over 2-fold, *P*≤0.05) in imbibed after-ripened relative to dormant seeds (Figure 5B, Table S2) [42]. As no probeset representing *GA 3-OXIDASE* (*GA3ox*) genes was present in wheat GeneChip, we analysed the expression of *TaGA3ox2* by qPCR. Our data showed no differential expression in *TaGA3ox2* between the dry dormant and after-ripened seeds; however, 14- to 52-fold more expression (*P*≤0.05) of this gene was apparent in imbibing after-ripened than dormant seeds (Figure 5C). The level of bioactive GAs in seeds is also regulated by its catabolism [81]. Previous studies have shown upregulation of *GA2ox2*, one of the GA inactivating genes, in imbibing dormant relative to after-ripened seeds of Arabidopsis [82]; however, all the *GA2ox* probesets exhibited almost identical expression between imbibing dormant and after-ripened wheat seeds (Figure 5B, Table S2). This result along with the upregulation of *GA20ox1* and *GA3ox2* genes in after-ripened seeds may suggest the role for after-ripening in increasing seed GA level during imbibition, and thereby enhancing seed dormancy decay and subsequent germination [61]. Contrary to this result, seed GA level has been shown not to be associated with after-ripening mediated seed dormancy decay in barley [11], [21]. Unfortunately, we could not explain our gene expression data in terms of seed GA level, as its concentration was below the detection limit of our system.

### Responsiveness of Wheat Seeds to GA Appears not to be Regulated Transcriptionally

**Figure 6:**
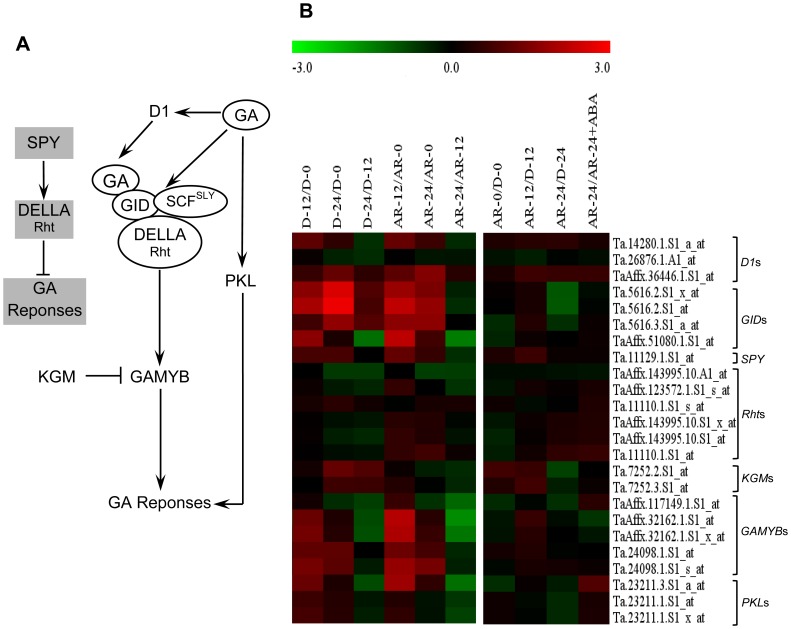
Comparison of the transcript abundance of gibberellin (GA) signaling genes. Molecular model for GA signaling pathways in plants (A). Changes in expression of probesets annotated as GA signaling genes in log_2_ fold change during imbibition of dormant (D-12/D-0, D-24/D-0 and D-24/D-0) and after-ripened (AR-12/AR-0, AR-24/AR-0 and AR-24/AR-0) seeds as shown in the first column of the heat map, between dormant and after-ripened seeds in both dry and imbibed states (AR-0/D-0, AR-12/D-12 and AR-24/D-24) and between water and ABA imbibed after-ripened seeds (AR-24/AR-24+ABA) as shown in the second column in each heat map (B). Determination of the fold changes in expression of each probeset is as described in Figure 2. Log_2_ and linear scaled fold changes in expression of the probesets and the respective *P* values can be found in Table S2. GPA, G protein α-sub unit; GID, GA insensitive dwarf; SCF; Skp1-cullin-F-box; SLY, sleepy1; Rht, reduced height; GAMYB, GA-regulated MYB transcription factor; KGM; kinase associated with GAMYB; PKL, pickel; SPY, spindly.

Several GA signaling factors are involved in regulating seed germination [24]. Twenty four probesets annotated as eight GA signaling genes (Figure 6A) are present on the wheat GeneChip, and all of them exhibited similar expression between dry dormant and after-ripened seeds (Figure 6B, Table S2). The action of GA takes place through GID1, a soluble GA receptor protein, and all the four probesets annotated as *GID1* exhibited either similar expression or downregulation (2-fold, *P*≤0.05) in imbibing after-ripened relative to dormant seeds. This result might reflect that GA signaling in these seeds is not dependent on GID1 levels. Similarly, mutation in *GID1* did not inhibit the germination of rice seeds [22], although it repressed the synthesis of α-amylase. However, probesets annotated as GA responsive genes such as those encoding amylases and cell wall degrading enzymes exhibit upregulation in imbibing after-ripened seeds [42]. Furthermore, translation of wheat probesets upregulated specifically in imbibing after-ripened seeds (at fourfold cutoff and *P*≤0.05) into their Arabidopsis and barley equivalents by microarray platform translator (http://www.plexdb.org/modules/PD_general/tools.php) [45] identified 49 and 61 GA induced genes in imbibing Arabidopsis [83] and barley [84] seeds, respectively. These results suggest that *GID* genes represented by the four probesets do not participate in perceiving GA signal or are activated through posttranscriptional mechanisms. GA promotes seed germination via degradation of DELLA proteins, which repress GA activated responses [85] and enhance seed dormancy, for example, by inhibiting the expansion of cotyledons [86]. Similar to barley (*SLN1*) and rice (*SLENDER RICE1*, *SLR1*), wheat contains a single DELLA gene (*Reduced height*, *Rht*) [87], and the expression of all of the six probesets annotated as *RHT* was not affected by either after-ripening or imbibition (Figure 6B, Table S2). It is likely that the responsiveness of germinating wheat seeds to GA is subjected to posttranscriptional regulation of *Rht* or may take place through a DELLA independent pathway [88]. The expression of probesets representing other GA signaling components such as pickle (PKL), a chromatin remodelling factor mediating GA induced activation of germination by repressing embryonic traits [89], and α-subunit of G protein (D1), a homolog of *GPA1*, acting as a positive regulator of seed responsiveness to GA [90], was not altered by after-ripening (Figure 6B). Probesets annotated as *KINASE ASSOCIATED WITH GAMYB1* (*KGM1*) and *SPY*, negative regulators of GA signaling, and *GAMYB*, encoding a positive transcriptional regulator of GA responsive genes, also exhibited similar expression between imbibing dormant and after-ripened seeds. Consistent with this result, a loss of function mutation in rice *GAMYB* inhibited α-amylase expression but not germination [91]. It appears therefore that the wheat homologs of *PKL*, *D1*, *KGM1*, *SPY* and *GAMYB* represented by the respective probesets are subjected to posttranscriptional regulation or do not affect GA signaling during imbibition. It is also possible that we might have missed identifying some of the transcriptionally regulated probesets due to our very stringent blast search criteria (see Materials and Methods).

### After-ripening Activates the Transcription of Specific Jasmonate Biosynthesis and Signaling Genes

**Figure 7:**
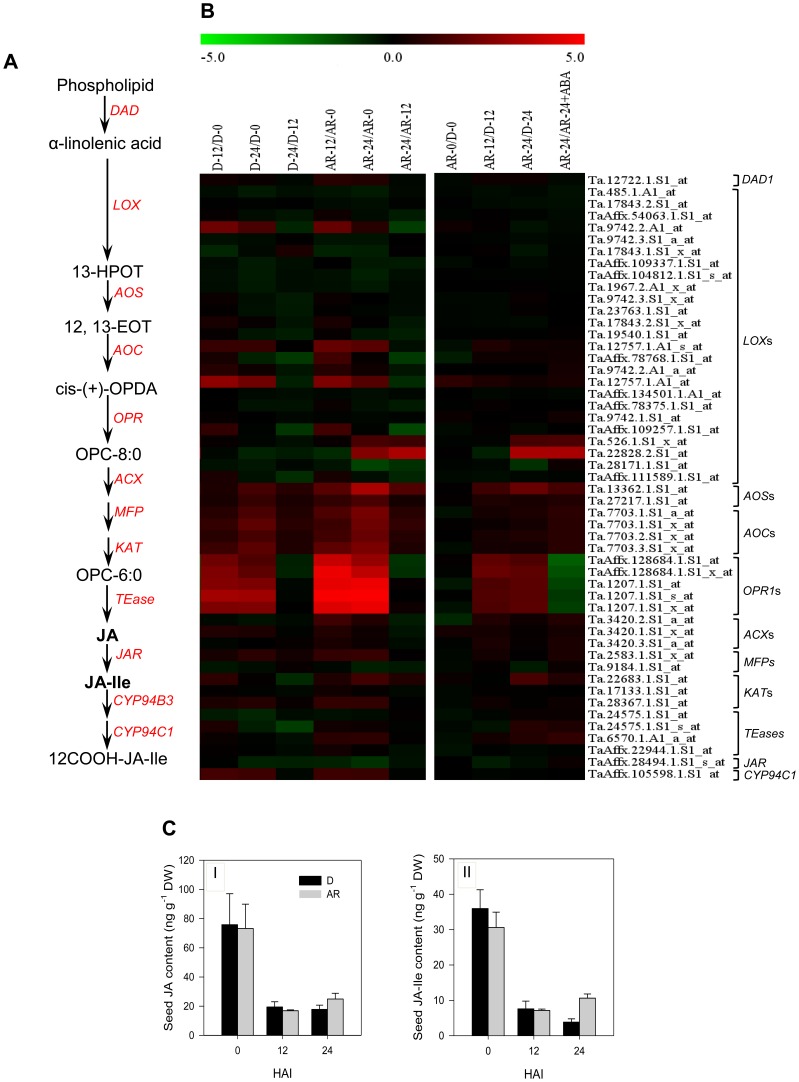
Transcript abundance of jasmonate metabolic genes, and seed jasmonic acid (JA) and JA-isoleucine (JA-Ile) content. Jasmonate metabolism pathway in plants (A). Expression of probesets annotated as jasmonate metabolic genes in log_2_ fold change during imbibition of dormant (D-12/D-0, D-24/D-0 and D-24/D-0) and after-ripened (AR-12/AR-0, AR-24/AR-0 and AR-24/AR-0) seeds as shown in the first column of the heat map, between dormant and after-ripened seeds in both dry and imbibed states (AR-0/D-0, AR-12/D-12 and AR-24/D-24) and between water and ABA imbibed after-ripened seeds (AR-24/AR-24+ABA) as shown in the second column in each heat map (B). Determination of the fold changes in expression of each probeset is as described in Figure 2. Log_2_ and linear scaled fold changes in expression of the probesets and the respective *P* values can be found in Table S2. JA and JA-Ile content of D and AR seeds in dry (0 days after imbibition [HAI]) and imbibed (12 and 24 HAI) states (C). Data are means of JA and JA-Ile measurements from three independent biological replicates ± SE. DAD, defender against cell death; LOX; lipoxygenase; 13-HPOT,13-hydroperoxylinolenic acid; AOS, allene oxide synthase; 12, 13-EOT, 12,13 epoxy-octadecatrienoic acid; AOC, allene oxide cyclase; cis-(+)-OPDA, cis-(+) -12-oxo phytodienoic acid; OPR3, 12-oxophytodienoate reductase; OPC-8∶0, 3-oxo-2-(2′-*Z*-pentenyl)-cyclopentane-1-octanoic acid; ACS, acyl-coenzyme A synthetase; ACX, acyl-coenzyme A oxidase; MFP, multifunctional protein; KAT, 3-ketoacyl coenzyme A thiolase; TEase; acyl-coenzyme A thioesterase; JAR, jasmonate resistant.

To gain better insight into the role of jasmonate in regulating seed dormancy and germination in wheat, we compared the expression of probesets annotated as jasmonate metabolic and signaling genes (Figure 7A), and the levels of JA and JA-Ile between dormant and after-ripened seeds in both dry and hydrated states. Analysis of our transcriptomic data with the criteria set (see Materials and Methods) revealed the presence of 55 probesets annotated as jasmonate metabolic genes, and all are expressed at similar level between dry dormant and after-ripened seeds (Figure 7B, Table S2). Consistently, there was no difference in seed JA and JA-Ile content between dry dormant and after-ripened samples (Figure 7C). After-ripening induced the transcriptional activation (2.3- to 10.0-fold, *P*≤0.05) of one probeset annotated as *AOS*, *3-KETOACYL COENZYME A THIOLASE3* (*KAT3*) and *LOX5* each, and five probesets annotated as *OPR1* during imbibition (Figure 7B, Table S2) [42]. Contrary to these gene expression results, seed imbibition was accompanied by a marked decline in the levels of JA and JA-Ile in both dormant and after-ripened seeds (Figure 7C). A recent study has identified two Arabidopsis genes, *CYP94B3* and *CYP94C1*, encoding enzymes that successively catalyze the conversion of bioactive JA-Ile to inactive 12OH-JA-Ile and then to 12COOH-JA-Ile, respectively [92]. Although no probeset representing *CYP94B3* was found with the search criteria we set, a probeset annotated as *CYP94C1* showed upregulation (2.2- to 2.5-fold, *P*≤0.05; Figure 7B) during imbibition irrespective of seed dormancy status, suggesting that seed JA/JA-Ile content during imbibition is closely associated with the expression of this jasmonate catabolic gene. However, the presence of approximately three-fold more JA-Ile in 24 h imbibed after-ripened than dormant seeds (Figure 7C) can be attributed to the after-ripening induced expression of jasmonate biosynthetic genes. Since JA-Ile but not OPDA, JA and methyl jasmonate enhance jasmonate signaling [93], our data imply that after-ripening regulates seed JA-Ile level, and thereby induces dormancy decay and germination. Consistently, increased expression of jasmonate biosynthetic genes and higher amounts of JA and JA-Ile were also evident in non-dormant than dormant seeds of Arabidopsis, although the amount decreased with imbibition [34]. Furthermore, after-ripening mediated upregulation of jasmonate biosynthetic genes was evident in the coleorhiza of imbibing barley seeds [11].

We also identified 24 probesets annotated as three JA signaling genes including *COI1*, *MITOGEN ACTIVATED PROTEIN KINASE6* (*MPK6*) and *MYC TRANSCRIPTION FACTOR2* (*MYC2*; Figure S1A). While the other jasmonate signaling probesets showed no differential expression between the two seed samples or exhibited upregulation (*P*≤0.05) in after-ripened as compared to dormant seeds during imbibition, a specific probeset annotated as *MPK* showed 2.4-fold downregulation (*P*≤0.05) in after-ripened relative to the corresponding dormant seeds following 24 h imbibition (Figure S1B, Table S2). Since MPK proteins inactivate JIN1/MYC2 that functions as an activator of jasmonate signaling, our data suggest that a specific *MPK* gene might be associated with activation of jasmonate response, and thereby seed dormancy decay and germination in wheat. This is supported by the transcriptional activation of jasmonate responsive genes including those involved in its biosynthesis such as *AOS* [20] in imbibing after-ripened relative to dormant seeds (Figure 7B, Table S2), and increased germination of dormant apple and *A. tataricum* seeds after treatment with exogenous JA [32]–[33].

### Seed IAA Level in Imbibing Wheat Seeds is Modulated by After-ripening

**Figure 8:**
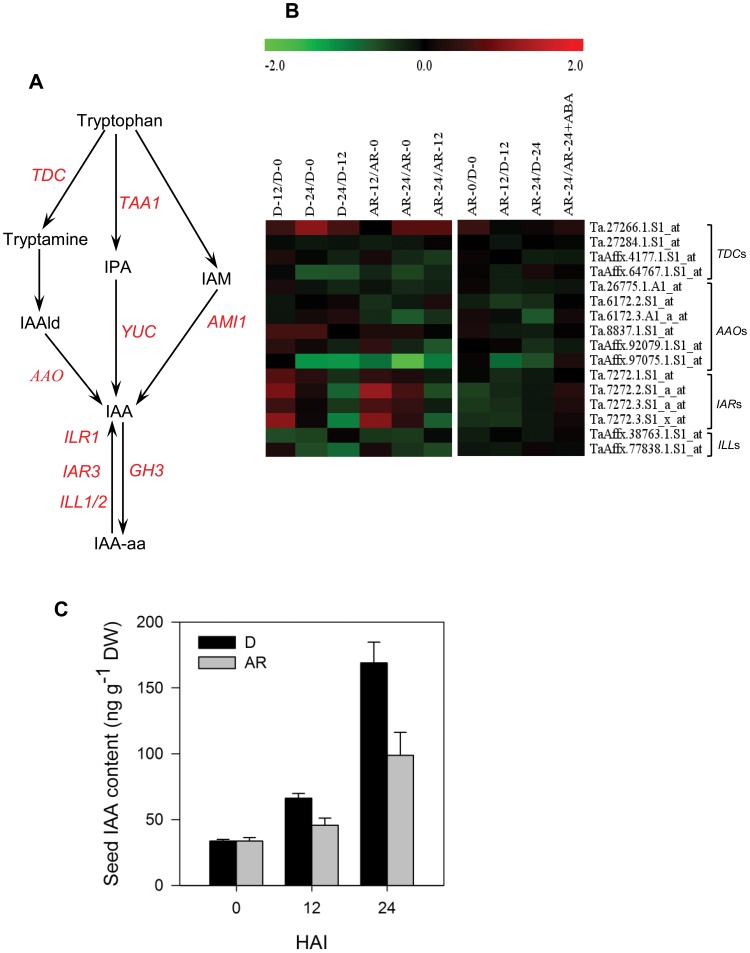
Comparison of the transcript abundance of IAA metabolic genes and seed IAA content. IAA metabolism pathway in plants excluding the Brassicaceae species specific pathway (A). Expression of probesets annotated as IAA metabolic genes in log_2_ fold change during imbibition of dormant (D-12/D-0, D-24/D-0 and D-24/D-0) and after-ripened (AR-12/AR-0, AR-24/AR-0 and AR-24/AR-0) seeds as shown in the first column of the heat map, between dormant and after-ripened seeds in both dry and imbibed states (AR-0/D-0, AR-12/D-12 and AR-24/D-24) and between water and ABA imbibed after-ripened seeds (AR-24/AR-24+ABA) as shown in the second column in each heat map (B). Determination of the fold changes in expression of each probeset is as described in Figure 2. Log_2_ and linear scaled fold changes in expression of the probesets and the respective *P* values can be found in Table S2. IAA content of D and AR seeds in dry (0 days after imbibition [HAI]) and imbibed (12 and 24 HAI) states (C). Data are means of IAA measurements from three independent biological replicates ± SE. Trp, tryptophan; TAM, tryptamine; IAAld, indole-3-acetaldehyde; IPA, indole-3-pyruvic acid; IAA, indole-3-acetic acid; IAM, indole-3-acetamide; TAA, tryptophan aminotransferase; YUC, YUCCA; AMI1, indole-3-acetamide hydrolase; ILR, IAA-leucine resistant 1; IAR; IAA-alanine resistant; ILL; IAA-leucine resistant 1-like; TDC, tyrosine decarboxylase.

A total of 16 probesets representing IAA metabolic genes, excluding those involved specifically in Brassicaceae species, are present on the wheat GeneChip (Figure 8A). All these probesets exhibited similar expression between after-ripened and dormant seeds in both dry and hydrated states, except that one probeset annotated as *AAO* was downregulated (2.2- to 3.6-fold, *P*≤0.05) following 24 h imbibition in both seed samples (Figure 8B, Table S2). Amide-linked conjugates of IAA synthesized during seed development [94]–[95] also serve as source of free IAA during seed germination and seedling growth [96]–[98]. Genes encoding IAA-amino acid hydrolases that release free IAA from the conjugates such as *IAA-LEUCINE RESISTANT1* (*ILR1*), *IAA-ALANINE RESISTANT3* (*IAR3*), *IAA-LEUCINE RESISTANT 1-LIKE1* (*ILL1*) and *ILL2* have been identified in the model plant Arabidopsis [99]. Six probesets annotated as these genes showed no difference in their expression between dry after-ripened and dormant seeds (Figure 8B, Table S2). However, two of the four probesets representing *IAR3* were induced (2.0- to 2.3-fold, *P*≤0.05) during the first 12 h imbibition in both after-ripened and dormant seeds. This is well correlated with increased seed IAA content (3- to 5-fold) during imbibition of both dormant and after-ripened seeds (Figure 8C), suggesting that at least some of the free IAA detected in imbibing seeds is hydrolyzed from IAA conjugates. The induction of seed IAA level during imbibition suggests that certain amount of seed auxin is necessary, if not essential, to initiate the germination of wheat seeds. Similarly, up-regulation of several genes related to auxin production and increased IAA level was observed during seed imbibition in Arabidopsis [34], and germination and seedling growth in pea [37]. However, imbibing after-ripened seeds contained only 58% to 69% of the IAA detected in the corresponding dormant seeds (Figure 8C). As auxin induces hypersensitivity of seeds to ABA and thereby inhibit germination [31], [38], our result may suggest that after-ripening induced dormancy release in wheat seeds is mediated partly by modulation of seed IAA content.

### After-ripening Induces Transcriptional Repression of Specific Auxin Signaling Genes

**Figure 9:**
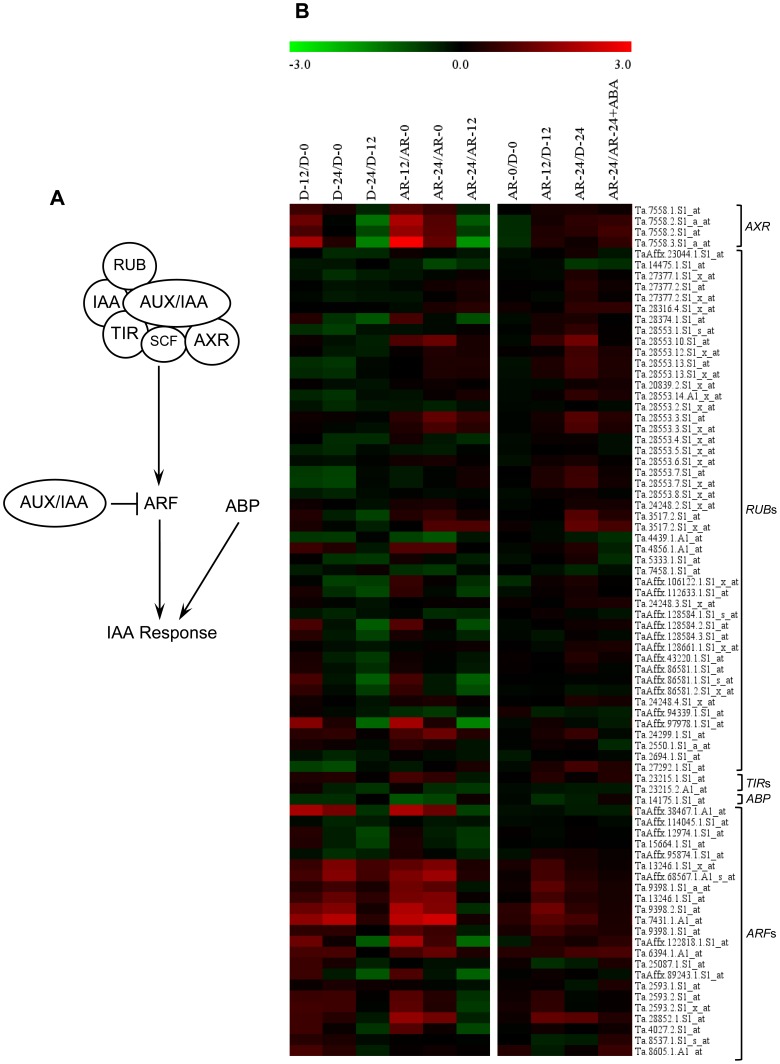
Comparison of the transcript abundance of auxin signaling genes. Molecular model for IAA signaling pathways in plants (A). Expression of probesets annotated as IAA signaling genes in log_2_ fold change during imbibition of dormant (D-12/D-0, D-24/D-0 and D-24/D-0) and after-ripened (AR-12/AR-0, AR-24/AR-0 and AR-24/AR-0) seeds as shown in the first column of the heat map, between dormant and after-ripened seeds in both dry and imbibed states (AR-0/D-0, AR-12/D-12 and AR-24/D-24) and between water and ABA imbibed after-ripened seeds (AR-24/AR-24+ABA) as shown in the second column in each heat map (B). Determination of the fold changes in expression of each probeset is as described in Figure 2. Log_2_ and linear scaled fold changes in expression of the probesets and the respective *P* values can be found in Table S2. TIR, transport inhibitor response; AXR; auxin-resistant; Aux/IAA, auxin/indole-3-acetic acid; RUB, related to ubiquitin; SCF; Skp1-cullin-F-box; ARF, auxin response factor; ABP, auxin binding protein.

To understand the role of auxin signaling factors in regulating the release of seed dormancy and germination in wheat, the expression of 78 probesets annotated as five auxin signaling genes, including *AUXIN-RESISTANT1* (*AXR1*), *UBIQUITIN-RELATED PROTEIN1* (*RUB1*), *TRANSPORT INHIBITOR RESPONSE1* (*TIR1*), *AUXIN-RESPONSE FACTOR* (*ARF*) and *AUXIN-BINDING PROTEIN1* (*ABP1*) were compared between after-ripened and dormant seed samples. Four of the 23 probesets annotated as *ARF* and four of the 48 probesets representing *RUB1* were upregulated (2.0- to 2.5-fold, *P*≤0.05) in imbibed after-ripened relative to dormant seeds (Figure 9B, Table S2). A total of four probesets were annotated as *AXR1*, encoding ubiquitin activating enzyme E1, and two of them exhibited upregulation only in imbibing after-ripened seeds (2.1- to 3.4-fold, *P*≤0.05). The AXR1 protein is associated with proteasome-mediated degradation of AUX/IAA [100], which inhibits ARF that acts as either transcriptional activator or repressor of auxin signalling [101]. As the specific *ARF* probeset that exhibited upregulation specifically in imbibing after-ripened wheat seeds was annotated as *ARF2* of Arabidopsis, which has been suggested as a repressor of cell division and organ growth [102]–[103], our data might suggest that one mechanism by which after-ripening mediates dormancy release and germination in wheat seeds is through repression of auxin signaling. Consistently, auxin insensitive mutants exhibit reduced dormancy [39] and exogenous auxin induces ABA hypersensitivity in germinating seeds [31], [38]. Since the ABI3 protein is postulated to act downstream of auxin [104], it is likely that decreased auxin signaling leads to suppression of seed sensitivity to ABA. This hypothesis is further supported by the induction of probesets annotated as *RUB1* (gene encoding a protein related to ubiquitin, the cullin subunit of SCF) in imbibing after-ripened seeds. Covalent conjugation of RUB1 to the SCF subunit controls the activity of TIR1-containing SCF-complex [105], which acts as the core regulator of proteasome-mediated AUX/IAA degradation [106]. As RUB1 deconjugation has been reported to be critical for proper operation of auxin signaling [107], the upregulation of specific *RUB1* probesets in imbibing after-ripened seeds may suggest repression of auxin signaling and thereby seed sensitivity to ABA.

### Regulation of Plant Hormone Related Probesets by ABA

ABA treatment affected the expression of probesets representing hormone metabolism and signaling genes. It activated the transcription of probesets annotated as *ABA2*, *GA2ox1* and *OPR1* (2.1- to 3.6-fold, *P*≤0.05) but repressed that of *ABF3*, *AOS1*, and *LOX2* and *LOX5* probesets (2.1- to 10.0-fold, *P*≤0.05; Table S2). Changes in the expression of probesets annotated as GA and jasmonate metabolic genes implicate these hormones in enhancing seed germination [42]. Our result, therefore, may suggest that ABA induced repression of wheat seed germination and seminal root growth is mediated partly by activation of GA catabolism and suppression of jasmonate production. In contrast with this, the expression of five probesets annotated as *OPR1* increased in response to ABA treatment, and this might imply feedback regulation of *OPR1* in seeds. Although ABA is required for full activation of ABF3 [108], the repression of *ABF3* in response to ABA treatment might suggest that ABF3 does not regulate ABA signaling in wheat seeds.

Although whole seed samples are used for this study, 70% of the probesets regulated by after-ripening are shared by those differentially expressed between dormant and non-dormant wheat embryos [72] and over 96% of ABA produced by mature cereal seeds is derived from the embryo [7], reflecting the functional significance of the embryo with respect to after-ripened mediated changes in hormone metabolism and signaling, and seed responsiveness to ABA.

In conclusion, after-ripening mediated seed dormancy release in wheat appears to be associated with a decrease in ABA signaling with no effect on its metabolism. This reduction in seed ABA sensitivity leads to derepression/repression of ABA controlled biological processes, including chromatin assembly, cell wall loosening and GA catabolism, and thereby dormancy decay. Since auxin has previously been implicated in inducing seed hypersensitivity to ABA, the reduced ABA signaling can also be attributed to after-ripening induced modulation of seed IAA content and repression of auxin signaling. Furthermore, our data suggest that regulation of seed jasmonate content and activation of GA synthesis form an integral part of the mechanisms underlying the role of after-ripening in inducing seed dormancy breakage and germination in wheat. Given that seed dormancy release is often correlated with changes in seed hormone content and/or sensitivity, and PHS, which causes substantial losses in yield and quality of cereal crops, is closely associated with seed dormancy, the results of this study advances our understanding of the molecular mechanisms regulating seed dormancy in cereals, which is a prerequisite to develop molecular tools for improving PHS tolerance.

## Supporting Information

Figure S1 Comparison of the transcript abundance of jasmonate signaling genes. Molecular model for jasmonate signaling pathways in plants (A). Expression of probesets annotated as jasmonate signaling genes in log_2_ fold change during imbibition of dormant (D-12/D-0, D-24/D-0 and D-24/D-0) and after-ripened (AR-12/AR-0, AR-24/AR-0 and AR-24/AR-0) seeds as shown in the first column of the heat map, between dormant and after-ripened seeds in both dry and imbibed states (AR-0/D-0, AR-12/D-12 and AR-24/D-24) and between water and ABA imbibed after-ripened seeds (AR-24/AR-24+ABA) as shown in the second column in each heat map (B). Determination of the fold changes in expression of each probeset is as described in Figure 2. Log_2_ and linear scaled fold changes in expression of the probesets and the respective *P* values can be found in Table S2. COI, coronatine insensitive 1; JAZ; jasmonate ZIM-domain proteins; MPK6; mitogen activated protein kinase 6; JIN/MYC2, jasmonate insensitive 1/MYC transcription factor 2.
(TIF)

Table S1 Log transformed signal intensity of hormone metabolism and signaling related probesets in dormant and after-ripened seeds in both dry and imbibed states.
(XLSX)

Table S2 Expression values of hormone metabolism and signaling related probesets in both logarithmically (base 2) and linear scaled fold changes in dormant and after-ripened seeds.
(XLSX)

Table S3 Annotation of ABA controlled probesets differentially regulated by after-ripening, and enriched ontological categories.
(XLSX)

Table S4 Annotation of probesets differentially regulated by after-ripening independent of ABA and enriched ontological categories.
(XLSX)

Table S5 Probesets related to specific biological processes differentially regulated by ABA (Kappa-View based analysis).
(XLSX)
