# Supplementary figures and images for: Regulation of Wheat Seed Dormancy by After-Ripening Is Mediated by Specific Transcriptional Switches That Induce Changes in Seed Hormone Metabolism and Signaling
Source: PLoS One. 2013 Feb 20;8(2):e56570. doi: 10.1371/journal.pone.0056570 (PMC3577873; doi:10.1371/journal.pone.0056570)

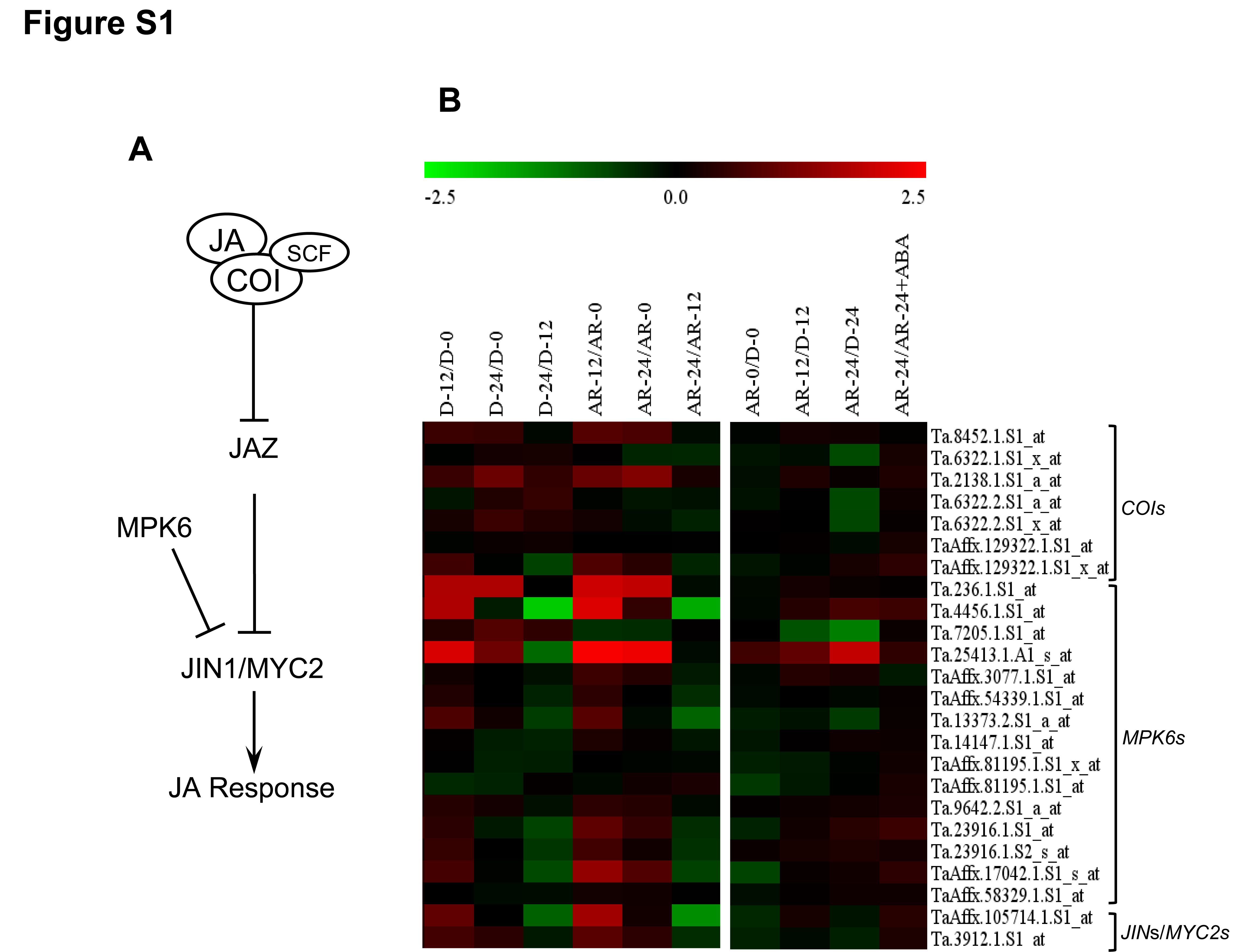

Supplement: Figure S1 — Comparison of the transcript abundance of jasmonate signaling genes. Molecular model for jasmonate signaling pathways in plants (A). Expression of probesets annotated as jasmonate signaling genes in log2 fold change during imbibition of dormant (D-12/D-0, D-24/D-0 and D-24/D-0) and after-ripened (AR-12/AR-0, AR-24/AR-0 and AR-24/AR-0) seeds as shown in the first column of the heat map, between dormant and after-ripened seeds in both dry and imbibed states (AR-0/D-0, AR-12/D-12 and AR-24/D-24) and between water and ABA imbibed after-ripened seeds (AR-24/AR-24+ABA) as shown in the second column in each heat map (B). Determination of the fold changes in expression of each probeset is as described in Figure 2. Log2 and linear scaled fold changes in expression of the probesets and the respective P values can be found in Table S2. COI, coronatine insensitive 1; JAZ; jasmonate ZIM-domain proteins; MPK6; mitogen activated protein kinase 6; JIN/MYC2, jasmonate insensitive 1/MYC transcription factor 2. (TIF) [file pone.0056570.s001.tif]
